# Transcriptomics-guided discovery of Interleukin-6 modulators from *Bacillus subtilis* metabolites in type 2 diabetes mellitus

**DOI:** 10.3389/fbinf.2026.1792877

**Published:** 2026-05-04

**Authors:** Tarsha Muthukumar, Sidharth Kumar Nanda Kumar, Vasundra Vasudevan, Madhana Priya Nanda Kumar, Thirumal Kumar D., Amudha Govindarajan, Magesh Ramasamy

**Affiliations:** 1 Department of Biotechnology, Faculty of Biomedical Sciences and Technology, Sri Ramachandra Institute of Higher Education and Research (DU), Chennai, India; 2 Department of Pharmacology, Center for Transdisciplinary Research, Saveetha Dental College and Hospitals, Saveetha Institute of Medical and Technical Sciences; 3 Meenakshi Academy of Higher Education and Research, Chennai, Tamil Nadu, India; 4 Department of Pharmacology, Government Medical College, Tiruvallur, India

**Keywords:** *Bacillus subtilis* metabolites, healthcare, IL6, molecular dynamics simulation, transcriptomics, type 2 diabetes mellitus

## Abstract

Type 2 diabetes mellitus (T2DM) is characterized by chronic metabolic dysfunction and low-grade inflammation. This study aimed to identify inflammation-associated molecular targets in T2DM and to computationally evaluate the therapeutic potential of *Bacillus subtilis*–derived metabolites targeting the key inflammatory cytokine IL6. Publicly available human RNA-sequencing datasets were retrieved from the NCBI Gene Expression Omnibus and analyzed using GEO2R to compare lean, obese, and T2DM groups. Common differentially expressed genes (DEGs) were identified and functionally enriched, with IL6 prioritized as a central inflammatory target. The IL6 protein structure was prepared for structure-based screening. Fifty-five *B. subtilis* metabolites were screened using PyRx, followed by ADME and toxicity prediction. Top-ranked compounds were further evaluated using molecular docking and 500 ns molecular dynamics simulations, with metformin as a reference. Free energy landscape (FEL) and dynamic cross-correlation matrix (DCCM) analyses assessed ligand-induced conformational stability and internal protein dynamics. A total of 179 common DEGs were identified, enriched in cytokine-mediated inflammatory pathways out of which IL6 emerged as a consistently upregulated hub gene. Three metabolites showed favorable pharmacokinetics, low predicted toxicity, and stronger binding affinities to IL6 than metformin. Docking revealed stable interactions with key IL6 residues, while molecular dynamics confirmed sustained complex stability. FEL and dynamic cross-correlation matrix analyses showed ligand-dependent differences in conformational stability while preserving internal dynamics. This integrative transcriptomics and structure-based analysis highlights *B. subtilis* metabolites as computationally predicted IL6-binding compounds involved in T2DM-associated inflammation, identifying them as promising candidates for further investigation towards potential healthcare and therapeutic applications.

## 1 Introduction

Diabetes mellitus (DM) is a chronic metabolic disorder characterized by persistent hyperglycemia of which 90% of the cases accounts for Type 2 Diabetes Mellitus (T2DM). In T2DM, the response to insulin is highly lowered, and this condition is termed as insulin resistance (Goyal et al., 2023). It is caused by a combination of two main factors: defective secretion of insulin by pancreatic β-cells and inability of tissues to respond to insulin action. Patients with T2DM have a higher body fat percentage (obese) and are found to be predominantly distributed in the abdominal region (Galicia-Garcia et al., 2020). T2DM can affect the functioning of almost all organs in the body in the long run and this can include macroangiopathy, diabetic retinopathy, nephropathy, neuropathy, diabetic foot and increased vulnerability to infections, myopathy (Farmaki et al., 2020). Effective control of T2DM is necessary to prevent disease progression and also to reduce its acute and chronic complications. Therefore, early diagnosis and comprehensive management strategies targeting both glucose homeostasis and underlying inflammatory conditions are required for improving patient outcomes, their quality of life and also for long-term disease prognosis.

Interleukin-6 (IL-6) is a proinflammatory cytokine that plays a major role in the pathogenesis of T2DM. It can induce the development of insulin resistance as it can cause inflammation by controlling differentiation, migration, proliferation and cell apoptosis (Bilal et al., 2014). Consistent elevation of IL-6 has been associated with insulin resistance, β-cell dysfunction, and also metabolic dysregulation by activation of downstream signalling pathways which includes JAK/STAT and MAPK cascades. The current therapeutic strategies target the IL-6 receptor (IL-6R) or the gp-130 mediated signalling complex using monoclonal antibodies which inhibits receptor activation. Inhibition at the receptor-level has reflected clinical relevance in inflammatory disorders, but direct modulation of IL-6 remains comparatively underexplored.

Prospective observational studies were done and revealed that circulating IL-6 posed a higher risk of developing T2DM in the future (Jin et al., 2024). However, the centrality of IL6 within the broader transcriptomic picture of T2DM, and its potential as an effective therapeutic target, remains to be systematically examined using integrative omics-based approaches.

There is growing interest towards safe, orally administered compounds that are bioactive which can modulate inflammatory pathways in various metabolic disorders. Although many synthetic anti-inflammatory and anti-diabetic drugs are available, their long time use is being associated with problems like resistance and lower efficacy in inflammation-driven metabolic dysfunction (Gothai et al., 2016). Use of naturally obtained bioactive compounds particularly those originating from microorganisms and probiotics offer multiple advantages of having multifunctional therapeutic potential, oral safety and biocompatibility. These compounds have also shown to reduce the destructive effects of inflammation (Benameur et al., 2023). Identifying and evaluating these bioactive compounds may contribute in developing comprehensive and sustainable strategies for reducing inflammation, improving metabolic homeostasis and long term management of the disease condition (Alvarez-Leite, 2025).

*Bacillus subtilis* is a gram positive, spore forming bacteria and it has gained attention because of its probiotic potential and ability to produce a wide range of secondary metabolites (Alvarez-Leite, 2025; Iqbal et al., 2023). It has become a major workhorse in the field of biotechnology as it produces a wide range of commercially interesting products (Stülke et al., 2023). So *B. subtilis* metabolome can be used as a promising source for candidate molecules that can be used against inflammatory pathways associated with T2DM.

Structure-based molecular approaches provide a powerful framework for prioritizing candidate molecules prior to experimental validation. Molecular docking can be used to screen a library of metabolites against a target protein, to identify their binding patterns and interaction strengths. It can be used as a model to study the interaction of small molecules to the binding site of target proteins as well as to interpret biochemical processes. The overall docking process involves two main steps, which includes predicting the ligand conformation, orientation within the target sites and assessing their binding affinities (Meng et al., 2011). Furthermore, Molecular Dynamics, FEL and DCCM analysis provides deeper insights on the stability and dynamic interactions between the ligand and target (Ligand-protein complex) over time.

This study involves a transcriptomics-driven strategy to identify IL-6 as a central target in T2DM using the GEO databases that are publicly available. After target identification, a structure-based virtual screening of metabolites selected from *B. subtilis* against IL-6 was implemented. Initially, all compounds were subjected to virtual screening based on binding affinity and interaction profiles. The top-ranked ligands were further evaluated based on docking scores, binding interactions, and pharmacological relevance. From this, a subset of promising candidates were selected for molecular dynamics (MD) simulations to assess the stability of protein–ligand complexes over time. Metformin was included as a reference control for comparative analysis. This integrated approach aims to describe the transcriptomic relevance of IL-6 in T2DM and identify metabolites from *B. subtilis*, while assessing the dynamic and structural suitability of the screened candidates in context of inflammation associated with T2DM.

## 2 Methodology

### 2.1 Dataset retrieval

The NCBI Gene Expression Omnibus (GEO) database (Edgar et al., 2002) was used to retrieve transcriptomic databases related to T2DM. GEO is a database supported by the National Center for Biotechnology Information (NCBI) at the National Library of Medicine (NLM) that accepts raw and processed data with written descriptions of experimental design, sample attributes, and methodology for studies of high-throughput gene expression and genomics. Type 2 diabetes mellitus was the major keyword that was used to search for our data among the GEO databases. Additionally filters were used to restrict our search to *Homo sapiens* under organisms and Expression profiling by high throughput sequencing under study type. Only those data specific to epicardial adipose tissue were selected (Bioproject ID: PRJNA743878 https://www.ncbi.nlm.nih.gov/bioproject/PRJNA743878/, Accession number: GSE179455).

### 2.2 Differential gene expression analysis using GEO2R

The GEO2R tool (Clough et al., 2024) was used for differential gene expression analysis. It is an online tool that is used to compare two or more sample groups to identify genes that are differentially expressed across experimental conditions. GEO2R employs the limma (Linear Models for Microarray and RNA-seq Data) statistical framework for analyzing differential expression. The dataset was analyzed using the normalized expression values that have been log2-transformed and are included in the GEO Series matrix file. GEO2R carries out suitable normalization between samples to assess and adjust for technical variability, ensuring comparability among experimental groups. All samples came from the same tissue source, thus minimizing confounding effects associated with transcriptomic variation specific to organs. To reduce background noise and improve statistical robustness, low-expression probes were filtered out by removing genes with consistently low intensity values across all samples. Only genes meeting the defined expression threshold and showing sufficient variation were retained for downstream differential expression analysis. Batch effect correction was not explicitly performed, as GEO2R does not provide direct options for batch adjustment. While this approach ensures consistency and ease of analysis, it may not fully account for potential batch effects or other technical variations inherent in publicly available datasets. Therefore, residual confounding factors cannot be entirely excluded and should be considered when interpreting the results.

For each GSE selected, the samples were assigned to three groups on the basis of metabolic status which included lean (Group 1), Obese/Overweight (Group 2), T2DM (Group 3). Statistical analysis was made to compare Lean vs. T2DM (Group 1 vs. Group 3) and Lean vs. Obese (Group 1 vs. Group 2). Obesity is one of the major risk factors for T2DM and it represents a metabolic state which is characterised by inflammatory activation and also insulin resistance. Hence, genes differentially expressed in both Lean vs. Obese and Lean vs. T2DM comparisons are likely to present shared pathways that are involved in inflammatory signalling, metabolic stress and also in disease progression.

For each analysis, P value, log 2 fold Change were determined and computed. Criteria used for differentially expressed genes (DEGs) was |log2FC| ≥ 1 and adjusted p-value (FDR) ≤ 0.05. The expressed genes from both of the analyses were taken separately and this list was further used for downstream analyses. The common genes from these DEG sets were retrieved using Venny 2.1. It is a web-based bioinformatics software that was designed for comparing and visualising multiple datasets by constructing Venn diagrams.

### 2.3 Plotting and visualization

Multiple visualization approaches were made to understand the distribution pattern of the genes. To proceed, Gene enrichment (GO and KEGG pathways) analysis was done using SR plot. The KEGG enrichment (Kanehisa and Goto, 2000) was used to find out whether there was significant over-representation of DEGs in metabolic and signaling pathways associated with T2DM.The Kyoto Encyclopedia of Genes and Genomes (KEGG) is a publicly accessible comprehensive resource that combines genomic, systemic functional information for clear understanding of complex biological molecules. The Biological process (BP), Cellular component (CC) and Molecular function (MF) plots were obtained to select the biologically relevant genes that can be used for further analyses.

### 2.4 Protein retrieval and processing

The target protein selected for this study was Interleukin-6 (IL-6). For performing structure-based virtual screening and molecular docking, protein sequence and three-dimensional structure IL-6 were retrieved and prepared. The Uniport database was used to retrieve the canonical information including amino acid sequence, functional description of the protein. The 3D structure of IL-6 was obtained from the Protein Data Bank (PDB) (Berman et al., 2000). PDB is an international, publicly accessible repository that contains three-dimensional structural data of biological molecules including proteins, nucleic acids and protein-ligand complexes. The PDB file was downloaded (PDB ID:1ALU) and was used as an initial structure for further protein preparation. IL-6 was obtained in the monomeric form and the single polypeptide chain was selected after the removal of heteroatoms and water molecules to represent the receptor structure for ligand interaction analysis. Protonation states were assigned at physiological pH (7.4) using the default protein preparation protocol in AutoDock Tools, where polar hydrogens and Kollman charges were added. Although explicit pKa-based optimization was not performed, this approach is widely accepted for molecular docking studies focused on relative binding interactions.

### 2.5 Protein preparation

The retrieved IL-6 structure was opened in PYMOL (Schrödinger, 2020) for inspection and cleaning. The final structure was saved and used in the Computed Atlas of Surface Topography of Proteins for binding site prediction (CASTp) (Tian et al., 2018). The Computed Atlas of Protein Surface Topography (CASTpFold) was used to identify and measure concave surface regions on three-dimensional structure of the protein. In the CASTpFold tool the search icon was chosen and the PDB ID was used as the input. Together with the area and volume, the search result displays the residues that are located within the binding region of the protein. Energy minimization is essential for determining the proper molecule arrangement in space when the chemical structures that are drawn are not energetically advantageous. When an energy reduction algorithm is performed, it will rapidly approach a low local energy value since a molecule’s potential energy has multiple energy components, including stretching, bending, and torsion. The Swiss-PDBviewer (SPDBV) tool was used for Energy Minimization.

### 2.6 Ligand retrieval

For virtual screening against the prepared protein targets, potential small molecular ligands were collected. Secondary metabolites produced by the bacterium *B. subtilis* were listed out and checked for their positive effects on treatment of T2DM. A list of 55 metabolites was created and the 2D structures of these metabolites were retrieved from the pubchem databases. These structures were downloaded as SDF files and their smiles were also documented.

### 2.7 Virtual screening

The aim of virtual screening is to evaluate a large number of ligands against a target protein to find out promising hits. PyRx (Dallakyan and Olson, 2015) was used for the structure-based virtual screening where the prepared protein and the ligand library were used as inputs. All the ligands were docked to the target and binding scores were generated and evaluated. The docking results of each ligand were arranged according to their binding energies (Stronger binding-More negative values). Top-ranked ligands were chosen as preliminary hits and used for further ADME and toxicity Prediction.

### 2.8 ADME and toxicity prediction

To evaluate the pharmacokinetic and safety properties of the virtual hits, ADME and toxicity testing were done. For this SwissADME (Daina et al., 2017) and ADMETlab (Xiong et al., 2021) were used to which smiles of the virtual hits were submitted. SwissADME was used to determine key physicochemical parameters (molecular weight, Log P) as well as Pharmacokinetic properties including Absorption (Gastrointestinal absorption, skin permeability), Distribution (plasma protein binding, blood-brain barrier permeability), Metabolism (interactions with cytochrome p450 enzyme, potential inhibition/induction), Excretion (clearance, half life) were predicted. Furthermore, compliance with classical drug-likeness filters (e.g.,Lipinski) was also determined for favourable oral bioavailability.

Toxicity studies were done using Protox III (Banerjee et al., 2024) which included the risks of mutagenicity, hepatotoxicity and other adverse effects. Ligands with high predicted toxicity or poor ADME characteristics (very low predicted absorption, unfavorable lipophilicity) were not taken into further consideration.

### 2.9 Molecular docking

AutoDock software was used for carrying out molecular docking to investigate the binding interactions between the three ADME-filtered ligands and the IL-6 protein (Thirumal Kumar et al., 2022). The IL-6 structure in the PDBQT format was uploaded to the Autodock Tool and a grid box was formed around the binding pockets which were identified previously by CASTp and visualised in pyMOL. For molecular docking, the grid box was defined with dimensions of 52 × 52 × 92 grid points and a spacing of 0.375 Å. The grid center was set at coordinates x = 5.573, y = −19.992 and z = 9.058, ensuring adequate coverage of the active binding region of the target protein.

The search method used was Lamarckian Genetic Algorithm (LGA) with which docking was performed. For each ligand, Autodock generated binding poses and also their corresponding binding energy scores. For detailed post-docking analysis, the docking output files were imported into the Discovery Studio Visualiser. It generates high quality 2D and 3D interaction figures (Madhana Priya et al., 2024).

### 2.10 Molecular dynamics and post MD analysis

Molecular dynamics (MD) simulations were performed to evaluate the conformational stability and dynamic behavior of IL-6 in complex with the top-ranked metabolites Cis-Cyclo (3-chloro-Tyr-Ile), Digallate and the control drug metformin. All protein–ligand complexes obtained from docking were subjected to 500 ns all-atom MD simulations using GROMACS (Abraham et al., 2015) with CHARMM27 all-atom force field for the protein. The CHARMM27 force field was selected because of its reliable performance in reproducing protein structural dynamics and also its compatibility with ligand parameters generated through the SwissParam server. CHARMM27 remains a validated and widely used force field in biomolecular simulations, particularly for exploratory and comparative studies. In the current work, the primary goal was to find the relative stability and interaction patterns of protein-ligand complexes rather than to obtain absolute thermodynamics quantities. Hence, CHARMM27 was considered sufficient for assessing the essential conformational dynamics. GROMACS (Abraham et al., 2015) with CHARMM27 all-atom force field for the protein. The CHARMM27 force field was selected because of its reliable performance in reproducing protein structural dynamics and also its compatibility with ligand parameters generated through the SwissParam server.

The systems were solvated in a cubic box with explicit TIP3P water molecules and neutralized with counter ions. Energy minimization was carried out using the steepest descent algorithm, followed by equilibration under NVT and NPT ensembles to stabilize temperature (300 K) and pressure (1 bar), respectively (Priya et al., 2025). Long-range electrostatics were treated using the Particle Mesh Ewald method, and hydrogen bonds were constrained using the LINCS algorithm. A production run of 500 ns was executed with periodic boundary conditions. Post-simulation analyses including RMSD, RMSF, radius of gyration, hydrogen bond occupancy and PCA were conducted to assess complex stability and ligand retention within the IL-6 binding pocket (Priyanka et al., 2025).

### 2.11 Free energy landscape (FEL) analysis

Free Energy Landscape analysis (Maisuradze et al., 2010) was performed which was used to evaluate the dynamic behaviour and conformational stability of the ligand- IL-6 complexes during the 500 ns molecular dynamics simulations. Using principal component analysis (PCA) FEL plots were generated, where the first two principal components (PC1 and PC2) were selected as reaction coordinates to construct the FEL plots. Two-dimensional and three-dimensional FEL plots were generated to represent the distribution of free energy that corresponds to the most stable and conformational states of ligands within the binding pockets of IL-6.

### 2.12 Dynamic cross-correlation matrix analysis (DCCM)

DCCM analysis (Kasahara et al., 2014) was performed to find out the correlated and non-correlated motions of the amino acid residues that occur during the molecular dynamics simulations (MD). It helps in understanding the ligand binding effects on the internal dynamics and also the collective motions of the target protein. After the MD simulation, the trajectory files were processed further by removing periodic boundary conditions and by fitting the structures to a reference frame which eliminates overall rotational and translational movements. Further, DCCM calculations were made using the Cα atoms of the protein residues which was used in assessing the residue-residue motion that correlates throughout the simulation time.

## 3 Results

### 3.1 Dataset retrieval

For the transcriptomic analysis, a total of 19 RNA-sequence Runs were retrieved (Table 1) from the NCBI Platform which included transcriptomic analysis that comprised lean, obese/overweight and type 2 metabolic groups. All the runs were annotated as transcriptomic libraries and this distribution of runs across different metabolic groups helped in comparative evaluation of the alterations in gene expression that are associated with obesity and its progression to T2DM.

**TABLE 1:** Overview of RNA sequencing runs retrieved from the NCBI platform, representing run accession, library layout, library source, and corresponding metabolic status.

Run	LibraryLayout	LibrarySource	metabolic_status
SRR15043364	PAIRED	TRANSCRIPTOMIC	Lean
SRR15043365	PAIRED	TRANSCRIPTOMIC	Lean
SRR15043366	PAIRED	TRANSCRIPTOMIC	Lean
SRR15043367	PAIRED	TRANSCRIPTOMIC	Lean
SRR15043368	PAIRED	TRANSCRIPTOMIC	Lean
SRR15043362	PAIRED	TRANSCRIPTOMIC	Lean
SRR15043363	PAIRED	TRANSCRIPTOMIC	Lean
SRR15043369	PAIRED	TRANSCRIPTOMIC	Obese/overweight
SRR15043370	PAIRED	TRANSCRIPTOMIC	Obese/overweight
SRR15043371	PAIRED	TRANSCRIPTOMIC	Obese/overweight
SRR15043372	PAIRED	TRANSCRIPTOMIC	Obese/overweight
SRR15043373	PAIRED	TRANSCRIPTOMIC	Obese/overweight
SRR15043374	SINGLE	TRANSCRIPTOMIC	Obese/overweight
SRR15043375	SINGLE	TRANSCRIPTOMIC	Obese/overweight
SRR15043376	PAIRED	TRANSCRIPTOMIC	Type 2 diabetic
SRR15043377	PAIRED	TRANSCRIPTOMIC	Type 2 diabetic
SRR15043378	PAIRED	TRANSCRIPTOMIC	Type 2 diabetic
SRR15043379	PAIRED	TRANSCRIPTOMIC	Type 2 diabetic
SRR15043380	PAIRED	TRANSCRIPTOMIC	Type 2 diabetic

### 3.2 Differential gene expression analysis using GEO2R and visualization

**FIGURE 1:**
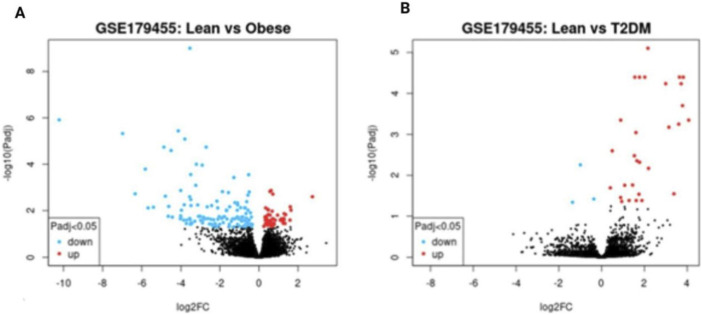
Volcano plot representing differentially expressed genes identified using GEO2R. Red dots indicate significantly upregulated genes and blue dots represent significantly downregulated genes (**A**- Lean vs. obese, **B**- Lean vs. T2DM).

**FIGURE 2:**
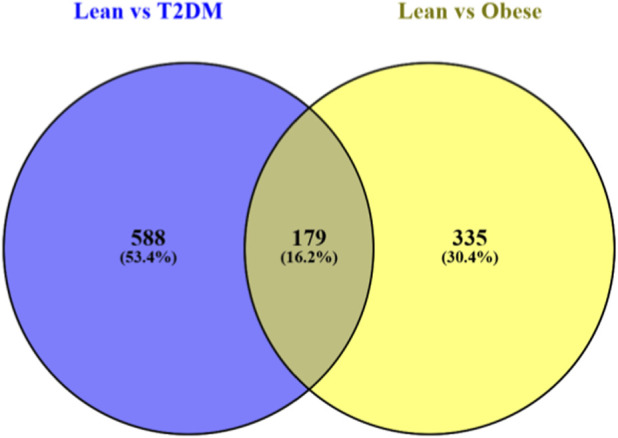
Venn diagram generated using Venny representing common genes between Lean vs. T2DM and Lean vs. Obese.

The Differential Gene Expression generated a list of genes showing significant differential expression between T2DM and the control samples. To obtain a refined set of genes that was statistically significant, these genes were filtered using adjusted p-values and log fold criteria. Both upregulated and downregulated genes were identified in the diabetic group of samples. Visualisation of these differential expressions was done using GEO-2R volcano plots and Bar plots (Figure 1). To identify commonly differentially expressed genes, the list of genes were analysed using Venny and a Venn diagram was obtained (Figure 2) representing overlapping genes shared between the two datasets.

A volcano plot is a graphical representation that is used in differential gene expression analysis to visualise changes between two conditions. The X-axis represents the magnitude and direction of the gene regulation and the Y-axis indicates the statistical significance. Genes that appear higher in the plot and farther from the center are found to be more biologically relevant and statistically significant. Hence, this visualisation helps in finding out key Differentially expressed genes that can be further used for downstream analysis, pathway enrichment and selection of targets on disease-based studies.

Venn diagrams created using Venny online tool are used in visualising the uniqueness and overlap of DEG’s that is obtained from multiple comparisons and databases. Each circle represents an individual dataset and the intersecting region represents common genes between the datasets. There were 588 DEG’s from lean vs. T2DM dataset and 335 DEG’s from Lean vs. obese dataset. A total of 179 genes which accounts for 16.2% were found common between the datasets (Figure 2).

The GO enrichment analysis provided an overview on biological processes, molecular activities and cellular localisation that were associated with the DEGs. In the Biological function (BP) category, enrichment was associated with immune response and regulation, metabolic processes and signal transduction. In the cellular component (CC) category, DEG’s were mainly localised in the extracellular region, cytoplasmic compartments and the plasma membrane. For the Molecular function (MF) category, enrichment was associated with receptor binding, cytokine activity and protein-protein interactions.

**FIGURE 3:**
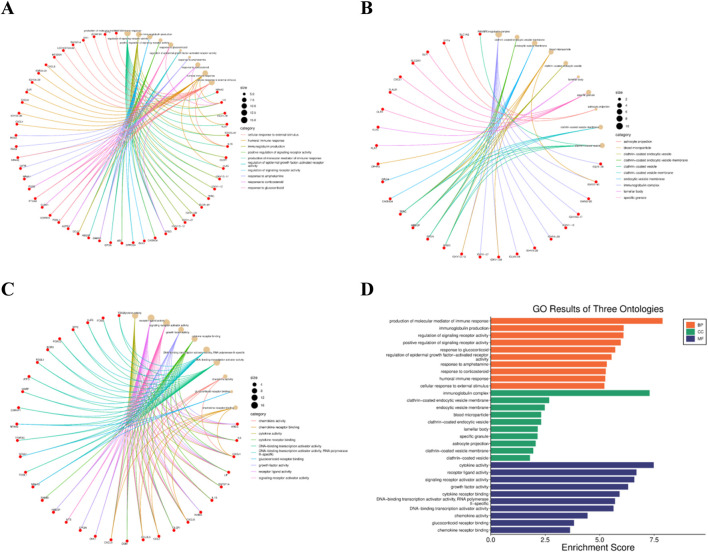
GO enrichment analysis of differentially expressed genes: **(A)** Biological Processes (BP), **(B)** Cellular Components (CC), and **(C)** Molecular Functions (MF) shown as enrichment networks, and **(D)** bar plot summarizing enriched GO terms across the three ontologies based on enrichment scores.

*IL6* was selected as the target gene (Figure 3) as it was observed that *IL6* was consistently and significantly upregulated in the differential gene expression analysis using GEO2R. *IL6* plays a major role in inflammation-mediated metabolic dysfunction which includes cytokine signaling, insulin resistance and immune response. Moreover, it functions as a pleiotropic cytokine that links chronic low-grade inflammation to impaired glucose homeostasis which is a hallmark of T2DM. Its involvement in key signaling pathways makes it a biologically suitable therapeutic target.

### 3.3 Protein and ligands structure retrieval

The three dimensional structure of the target protein IL-6 was retrieved in PDB format from the Protein Data Bank (PDB ID: 1ALU) (Somers et al., 1997). The structure was chosen based on suitability and resolution for accurate molecular docking results. Water molecules and other heteroatoms were identified and removed during the protein preparation stage. The removal of water molecules was done after visual inspection of the structure. No conserved or functionally annotated structural water molecules that directly mediate ligand–protein interactions in the IL-6 interface were identified.

Ligand structures were obtained from the PubChem database in SDF (Structure Data File) form. The structure of Metformin was also retrieved and it was used as a control for comparative analysis. All ligand structures were downloaded in appropriate format required for molecular docking.

### 3.4 Protein preparation

During the protein preparation and analysis of the binding-sites of IL-6, key interacting residues were identified within the predicted ligand-binding region. The final complex showed notable contacts with GLU95, VAL96, LEU98, GLU99, GLN116, LYS120, PRO141, and ASN144, representing that these residues contribute to effective stabilization of the ligand within the IL-6 binding pocket.

Following protein preparation, the IL-6 structure was energy minimised to eliminate steric clashes and for the optimisation of the local geometry prior to docking and molecular dynamics simulations. Post-minimisation showed that the overall fold of IL-6 was preserved, while minute adjustments were observed in the side-chain conformations of residues GLU95, VAL96, LEU98, GLU99, GLN116, LYS120, PRO141, and ASN144. These residues were located within the putative binding region and it adopted more favourable orientations, suggesting improved packing and stabilization of the local environment around the ligand-binding site.

The energy components included bond energy (220.972 kJ/mol), angle energy (533.416 kJ/mol), dihedral energy (790.590 kJ/mol), and improper interactions (227.892 kJ/mol), while significant negative contributions were observed from non-bonded interactions, including Coulombic (−5,074.78 kJ/mol) and van der Waals energies (−6,500.38 kJ/mol). The total potential energy of the system after minimization was found to be −9,802.289 kJ/mol, which indicates a thermodynamically stable configuration which is an absolute necessity for further investigation. Energy minimization ensures that the protein is relaxed into a local energy minimum and this preparatory step is crucial as it ensures structural reliability and prevents artificial instability during ligand binding and dynamic analysis.

### 3.5 Virtual screening, ADME and toxicity prediction

Following protein preparation, the optimized IL-6 structure was used for virtual screening of the metabolite library. The binding affinities of 55 metabolites from *Bacillus subtilis* against IL-6 were evaluated along with their ADME and toxicity profiles (Table 2).

**TABLE 2:** Representation of binding affinity, ADME and toxicity of the retrieved ligands.

Ligand	Binding affinity	SwissADME (lipinski rule of 5 violations)	ADMETLAB (drug likeness)	Protox III (toxicity)
il6_emp_77844231_uff_E = 597.95	−6.8	1 violation (NH or OH > 5)	PASS	4
il6_emp_24905973_uff_E = 1,040.26	−6.6	2 violation (N or O > 10, NH or OH > 5)	FAIL	4
il6_emp_24905928_uff_E = 967.07	−6.5	2 violation (N or O > 10, NH or OH > 5)	FAIL	4
il6_emp_77844233_uff_E = 859.22	−6.5	1 violation (NH or OH > 5)	PASS	4
il6_emp_156582882_uff_E = 1,258.87	−6.2	0 violation	PASS	3
il6_emp_139586981_uff_E = 604.64	−5.8	0 violation	PASS	4
il6_emp_170988961_uff_E = 267.29	−5.8	0 violation	PASS	5
il6_emp_86583338_uff_E = 1814.07	−5.7	0 violation	PASS	4
il6_emp_156582880_uff_E = 1970.12	−5.7	0 violation	PASS	3
il6_emp_15790238_uff_E = 321.31	−5.6	0 violation	PASS	4
il6_emp_54711004_uff_E = 195.39	−5.6	0 violation	PASS	5
il6_emp_139590858_uff_E = 1,046.49	−5.6	0 violation	PASS	4
il6_emp_139590859_uff_E = 1,037.31	−5.6	1 violation (MLOGP >4.15)	PASS	4
il6_emp_6453155_uff_E = 378.79	−5.5	0 violation	PASS	5
il6_emp_77844232_uff_E = 702.96	−5.5	1 violation (NH or OH > 5)	PASS	4

Among all the screened candidates, compounds il6_emp_170988961, il6_emp_54711004, and il6_emp_6453155 were highlighted based on docking performances and pharmacokinetic profiles that were favourable. The compounds showed a binding affinity of −5.8 kcal/mol, −5.6 kcal/mol, −5.5 kcal/mol with zero Lipinski violations and toxicity class 5. The absence of drug-likeness violations reflects favourable oral pharmacokinetic characteristics, while the toxicity class predicted indicates a comparatively lower toxicity risk profile within the computational model. Their selection shows a prioritization strategy which emphasizes a balanced pharmacokinetic suitability along with binding affinity.

Certain compounds were able to satisfy drug-likeness parameters, whereas others showed deviations from the optimal ranges which favoured oral bioavailability and pharmacokinetic suitability. Swiss ADME predictions provide key computational estimations which are based on the established molecular descriptors and it does not include evidence of *in vivo* bioavailability or clinical applicability. Hence, the results are interpreted as a screening tool in the early stage that was used to prioritize compounds for further investigation.

The compounds that failed ADMET screening (il6_emp_24905973 and il6_emp_24905928) showed multiple Lipinski’s rule violations (≥2), indicating poor drug-likeness and reduced oral bioavailability. Additionally, their ADMETLAB predictions flagged unfavorable pharmacokinetic and toxicity profiles, leading to their exclusion despite having moderate binding affinities.

The results of ADMET analysis for Cis-Cyclo (3-chloro-Tyr-Ile) demonstrated favorable physicochemical properties, including appropriate molecular weight, lipophilicity, and polarity. Absorption analysis reflected acceptable permeability, while distribution studies showed stable plasma protein binding and minimal blood–brain barrier penetration. Toxicity predictions indicated low cardiotoxicity and mutagenicity, with an overall acceptable safety profile, highlighting its potential as a promising candidate for further investigation.

### 3.6 Molecular docking

The docking results (Table 3) showed that Cis-Cyclo(3-chloro-Tyr-Ile) had a moderate binding affinity to IL-6 with the docking score of −7.01 kcal/mol. It was followed by Digallate with a score of −5.5 kcal/mol and Cyclo (histidyl-leucyl) at −5.24 kcal/mol. Among the tested ligands Cyclo (histidyl-leucyl) had the lowest binding affinity indicating lower interaction with IL-6 compared to the other two ligands. These values correlate to better binding affinity, which further indicates stable protein-ligand interactions within the binding regions. Hydrogen bonding and hydrophobic contacts in the interaction profile further reflect the structural compatibility of the selected metabolites with binding regions of IL-6. The control drug metformin (Ligand ID 4091) demonstrated the lowest binding affinity among the tested compounds, with an average docking score of −4.31 kcal/mol. Metformin was included as a reference ligand for comparative docking analysis. While metformin is not a direct IL-6 inhibitor, it has been reported to exert anti-inflammatory effects and modulate cytokine signaling pathways associated with IL-6 expression. In this study, metformin served primarily as a structural comparator to provide a baseline for interpreting docking scores and binding interactions of the screened compounds.

**TABLE 3:** Representation of Molecular docking results of different ligands and control with their ID and binding scores.

Ligand ID	Ligand	Score 1 (kcal/mol)	Score 2 (kcal/mol)	Average score (kcal/mol)
170988961	Cis-Cyclo(3-chloro-Tyr-Ile)	−7.01	−7.01	−7.01
54,711,004	Digallate	−5.5	−5.5	−5.5
6,453,155	Cyclo(histidyl-leucyl)	−5.24	−5.24	−5.24
Control				
4,091	Metformin	−4.31	−4.31	−4.31

**FIGURE 4:**
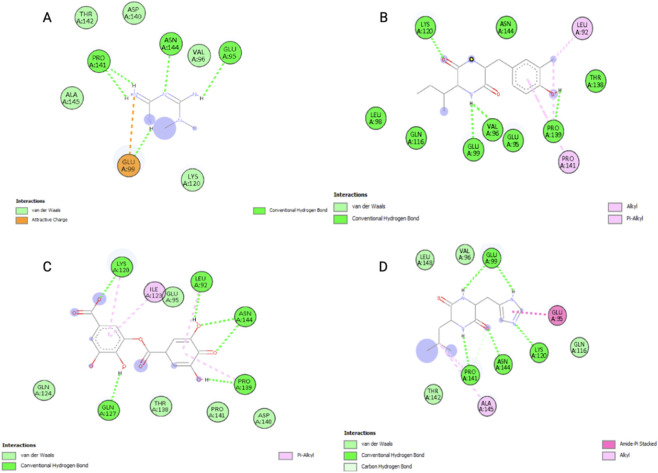
A 2D interaction diagram from the results of molecular docking-representing how different ligands interact with the binding sites of IL-6 [**A**-control, **B**-Cis-Cyclo (3-chloro-Tyr-Ile), **C**-Digallate, **D**-Cyclo (histidyl-leucyl)].

In all ligand-protein interaction, the ligands were held in place by typical hydrogen bonds and several van der Waals interactions with important residues like GLU A:95, GLU A:99, ASN A:144, LYS A:120, PRO A:141, and THR A:142 (Figure 4). Electrostatic (attractive charge) and amide-π stacked interactions further strengthen the ligand attachment. According to the results, ligands B and C exhibited large amounts of interactions, supporting their elevated docking affinity values. It is important to note that docking provides preliminary structural compatibility rather than evidence of potency or biological effect.

### 3.7 Molecular dynamics

**FIGURE 5:**
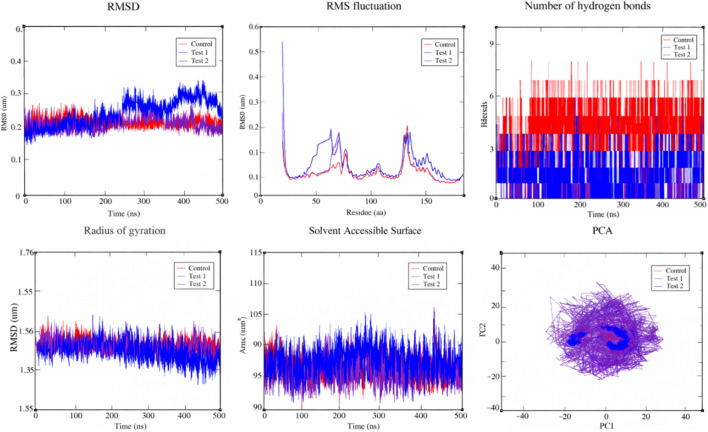
Molecular dynamics simulation analysis of the protein-ligand complex showing the conformational behaviour and stability over the simulation time.

The 500 ns molecular dynamics simulations were performed for the IL-6 complexes with Cis-Cyclo (3-chloro-Tyr-Ile), Digallate, and the control drug metformin to evaluate their dynamic stability and interaction behavior. The IL-6–Cis-Cyclo (3-chloro-Tyr-Ile) complex demonstrated stable RMSD values after equilibration with controlled fluctuations, reflecting adaptive binding accompanied by sustained pocket occupancy. RMSF analysis revealed reduced flexibility of critical residues, including GLU95, GLU99, GLN116, LYS120, PRO141, THR142, and ASN144, particularly in the Cis-Cyclo (3-chloro-Tyr-Ile) complex, indicating effective stabilization of the cytokine interface. The IL-6–Digallate complex showed moderate RMSD and RMSF variations, suggesting stable but less persistent binding relative to Cis-Cyclo (3-chloro-Tyr-Ile). Radius of gyration analysis confirmed maintenance of protein compactness in both metabolite-bound systems. Hydrogen bond analysis further demonstrated recurrent and long-lived interactions for Cis-Cyclo (3-chloro-Tyr-Ile) (Figure 5). It is important to note that subtle hydrogen bonding patterns and reduced RMSD values indicate structural accommodation of the ligand within the binding pocket; however, these parameters do not directly correlate with functional inhibition of IL-6 activity.

Although metformin displayed favorable RMSD behavior, its known gastrointestinal intolerance, lactic acidosis risk, and limited anti-inflammatory specificity reduce its suitability for chronic IL-6 modulation. In contrast, the *B. subtilis*–derived metabolites showed stable dynamic behavior, favorable interaction persistence, and predicted low toxicity, supporting their potential as safer modulators of IL-6–mediated inflammation in T2DM.

To quantitatively assess structural stability, key molecular dynamics parameters including RMSD, RMSF, radius of gyration (Rg), SASA, and hydrogen bonding were analyzed (Table 4). The backbone RMSD indicated stable trajectories for all systems, with Test 1 (0.197 ± 0.023 nm) showing slightly improved stability compared to the control (0.209 ± 0.016 nm), while Test 2 exhibited higher deviation (0.234 ± 0.043 nm). RMSF analysis revealed moderate residue flexibility in Test 1 (0.092 ± 0.052 nm) and higher fluctuations in Test 2 (0.114 ± 0.070 nm) compared to the control (0.079 ± 0.039 nm). Rg values were comparable across systems, indicating maintained compactness, with only minor variation in Test 2 (Table 4). SASA results showed similar solvent exposure for Test 1 and control, whereas Test 2 displayed increased surface accessibility. Hydrogen bond analysis indicated fewer interactions in Test 1 and Test 2 compared to the control; however, the lower RMSD and stable compactness of Test 1 suggest a stable binding configuration. Overall, Test 1 demonstrates stability comparable to or slightly better than the control, while Test 2 shows greater structural fluctuation and reduced stability.

**TABLE 4:** Statistical summary of molecular dynamics parameters including RMSD, RMSF, radius of gyration, SASA, and hydrogen bond occupancy for the control and test complexes. Values represent mean ± standard deviation calculated from the simulation trajectories.

Parameter	Control (mean ± SD)	Test 1 (mean ± SD)	Test 2 (mean ± SD)
RMSD (nm)	0.209 ± 0.016	0.197 ± 0.023	0.234 ± 0.043
RMSF (nm)	0.079 ± 0.039	0.092 ± 0.052	0.114 ± 0.070
Radius of gyration (nm)	1.582 ± 0.007	1.578 ± 0.008	1.573 ± 0.011
SASA (Å^2^)	9,495 ± 179	9,430 ± 215	9,648 ± 224
Hydrogen bonds	4.23 ± 1.04	0.71 ± 0.80	1.39 ± 0.97

### 3.8 Free energy landscape (FEL) analysis

**FIGURE 6:**
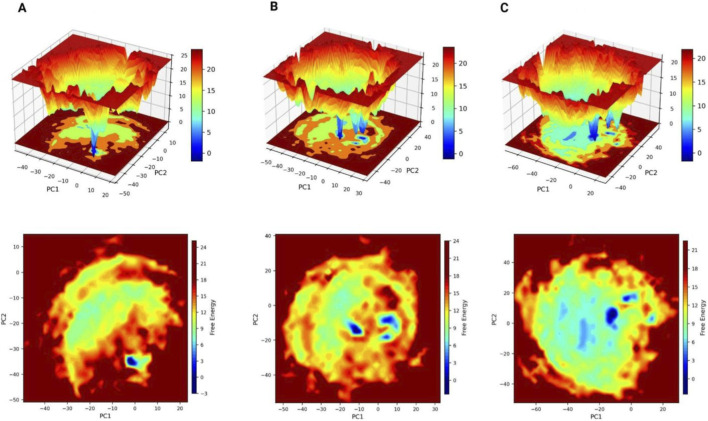
**(A–C)** 2D and 3D free energy landscape (FEL) plots of IL-6–ligand complexes constructed using the first two principal components (PC1 and PC2) during molecular dynamics simulations. **(A)** Control, **(B)** Test 1, and **(C)** Test 2 systems. For each panel, the top row represents the 3D FEL, and the bottom row shows the corresponding 2D FEL projection.

Free energy landscape (FEL) analysis revealed distinct differences in the conformational stability of the protein when complexed with the three ligands. The Cis-Cyclo (3-chloro-Tyr-Ile) complex displayed a compact and deep low-energy basin, indicating a highly confined conformational space and strong stabilization of the protein structure during the simulation. This pattern suggests that Cis-Cyclo (3-chloro-Tyr-Ile) effectively stabilizes the protein by maintaining it within a well-defined energetic minimum, reflecting stable ligand–protein interactions. In comparison, the Digallate complex exhibited two distinguishable low-energy minima, suggesting the presence of multiple stable conformational states and moderate structural stability. Conversely, the Metformin complex showed a broader and relatively shallow energy basin, indicating greater conformational flexibility and reduced restriction of the protein structure during the simulation (Figure 6). Overall, the conformational stability inferred from the FEL analysis follows the order: Cis-Cyclo (3-chloro-Tyr-Ile) > Digallate > Metformin. These simulations provide structural insights into conformational stability and ligand binding behaviour rather than direct evidence of functional inhibition. Therefore, the free energy landscape (FEL) results reflect the thermodynamic stability of the protein–ligand complex within the simulated environment.

### 3.9 Dynamic cross-correlation matrix analysis (DCCM)

**FIGURE 7:**
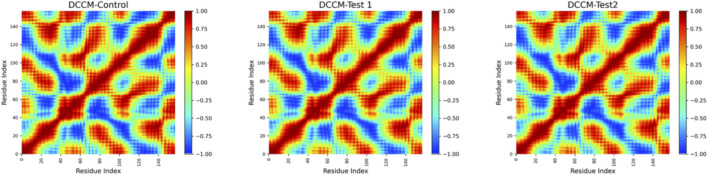
Dynamic Cross-Correlation Matrix Plots of IL-6 showing residue-wise correlated motions for the control, test one and test2 during molecular dynamics simulations.

In all the three systems, the control, Test1 and Test 2, the DCCM maps represented a similar global correlation pattern which was characterised by regions of positive (red) and negative (blue) correlation which were symmetrically distributed along the diagonal. In the control system, strong positive correlations were seen along the main diagonal, which reflected coherent motions among the neighbouring residues. Distinct blue regions were also observed representing anti-correlated motions between specific residue pairs (Figure 7).

The DCCM profiles of Test1, Test 2 were found to be closely resembling the control systems. This indicated that the overall dynamic behaviour of the protein backbone remains largely conserved on binding with the ligand. Overall, high similarity among the three profiles indicated that the ligands do not disturb the dynamic correlation nature of the protein and the protein maintains a stable, conserved dynamic behaviour across the control, test 1 and the test 2.

## 4 Discussion

T2DM is a metabolic disorder that is characterised by insulin resistance, chronic hyperglycemia and low grade systemic inflammation. Evidence shows that inflammatory mediators play a major role in the progression and complications of T2DM (Tsalamandris et al., 2019). An integrated silico-approach was employed in the present study to identify and characterize key molecular targets and potential small molecule modulators that are involved in the pathophysiology of T2DM, focusing particularly on inflammatory signaling pathways.

Gene expression analysis was performed initially using publicly available datasets obtained from the Gene Expression Omnibus (GEO). Differential expression analysis performed using GEO2R revealed that certain genes were dysregulated significantly between the diabetic and control groups. Most of the differentially expressed genes were associated with immune response, metabolic regulation and inflammatory pathways which correlated with previous transcriptomic studies in T2DM. Among all the genes identified, *IL6* was found to be a consistently upregulated gene across the datasets and this suggested its important role in disease progression. IL-6 is a Pleiotropic cytokine which plays a major role in glucose metabolism, insulin sensitivity and inflammatory responses (Dyatlova et al., 2023; Suzuki et al., 2011). In people with obesity and T2DM elevated circulating levels of IL-6 have been reported which provides strong evidence of association of IL-6 with insulin resistance and β-cell dysfunction. Chronic overexpression of IL-6 leads to sustained inflammation, which further exacerbates dysregulation of the metabolism. This consistent upregulation of IL-6 holds up its significance as a potential therapeutic target in T2DM. It is important to recognize that most clinically validated IL-6–directed therapies act at the receptor or pathway level rather than directly targeting the cytokine.

Pathway enrichment analysis performed using the KEGG database further showed the involvement of IL-6 associated signaling pathways in the mechanism of the disease. The enrichment pathways included cytokine-cytokine receptor interaction, inflammatory signaling cascades and metabolic regulatory networks. These pathways showed a strong interplay between inflammation and metabolic dysfunction in T2DM. All these findings further suggested that the modulation of the activity of IL-6 may help in restoring the metabolic balance and also to reduce the inflammatory burden of the disease.

Based on the observations made, IL-6 was chosen as the target protein for further molecular docking studies. The 3-dimensional structure of the protein was retrieved from the Protein Data Bank and it was prepared and optimised to get accurate docking results. Molecular docking is a structure-based drug discovery approach and it provides valuable information on protein-ligand interactions and also identifies potential modulators and inhibitors prior to experimental validations.

The ligand selection part was focused solely on the secondary metabolites derived from the bacterium *B. subtilis*. It is a bacterium known to produce a wide range of bioactive compounds that possess antimicrobial, non-inflammatory and therapeutic properties. These compounds were screened from the PubChem database and their toxicity, pharmacokinetic properties, drug-likeness were evaluated using SwissADME. This step was crucial to eliminate those compounds that had unfavourable absorption, distribution, metabolism, excretion, and toxicity profiles which helped in improving the likelihood of identifying viable drug candidates.

Toxicity analysis predicted that Metformin (control) showed a potential risk of gastrointestinal toxicity, associated with side effects including nausea, vomiting, diarrhea and abdominal discomfort. It was also associated with lactic acidosis which occurs under conditions of drug accumulation, which can be seen in cases of impaired renal function (Dyatlova et al., 2023).

Among all the screened compounds Cis-Cyclo (3-chloro-Tyr-Ile), Digallate and Cyclo (histidyl-leucyl) satisfied their safety as well as pharmacokinetic criteria and these were selected for further docking analysis. These metabolites represent potential bioactive candidates requiring further pharmacokinetic optimization and experimental validation. AutoDock was used for performing Molecular docking which revealed that Cis-Cyclo (3-chloro-Tyr-Ile) showed favourable binding affinity to IL-6. The ranking of ligands based on the average binding energy is summarized as:

$$\text{Cis}-\text{Cyclo }\left(3-\text{chloro}-\text{Tyr}-\text{Ile}\right)\;>\text{ Digallate }>\text{ Cyclo }\left(\text{histidyl}-\text{leucyl}\right)\;>\text{ Metformin}$$

The docking scores from the study were found to be between −5 and −7 kcal/mol, which reflects good binding affinity rather than a strong interaction. Therefore, these results should be interpreted as preliminary evidence of structural compatibility between the ligands rather than a confirmation of biological potency and therapeutic efficacy. Docking provides valuable insights into possible interactions and binding orientations within the target binding sites. Additionally, the comparison with metformin is presented only as a structural reference compound. Metformin is not a known IL-6 inhibitor therefore no pharmacological or functional equivalence is implied.

From the 2D interaction diagrams retrieved from the Discovery Studio, the top ranking ligands were found to form multiple hydrogen bonds and Wan der waals contacts with important residues including GLU A:95, GLU A:99, ASN A:144, LYS A:120, PRO A:141, and THR A:142. The consistency between the docking scores, and the strength of interactions indicates that these residues constitute an important pharmacophoric region within the binding pockets of the protein IL-6 Exploring IL-6 itself as a potential small-molecule target can provide complementary approaches for modulating IL-6–mediated inflammatory pathways. Structural analysis of IL-6 indicates the presence of surface regions that are involved in receptor recognition with residues particularly contributing to the IL-6/IL-6R interaction interface. In the present study, molecular docking results predicted ligand binding within a pocket that was located near these interaction regions, which suggests that ligand association may potentially influence receptor engagement by steric interference or localized conformational changes. However, it should be noted that these observations are derived based on computational modeling and do not yet confirm functional inhibition of IL-6 signaling. Therefore, the identified compounds should be considered as computationally predicted IL-6–binding compounds, and further experimental validation, including biophysical binding assays and *in vitro* functional studies, will be necessary to determine whether these interactions can effectively disrupt IL-6–IL-6R signaling.

Molecular dynamics simulations (Iqbal et al., 2025) provide critical insights to the study. *B. subtilis*–derived metabolites, particularly Cis-Cyclo (3-chloro-Tyr-Ile), demonstrated adaptive yet stable binding characterized by sustained pocket occupancy, reduced flexibility of functionally important residues, and persistent intermolecular hydrogen bonding throughout the 500 ns simulation. The RMSD, hydrogen bonding interactions, radius of gyration and solvent accessible surface area analysis indicate that the complexes remain structurally stable through the period of simulation. These parameters reflect the structural stability and accommodation of the ligands within the IL-6 binding pocket rather than providing evidence on functional inhibition.

The free energy profiles show differences in the extent of conformational confinement and the DCCM maps show that the overall correlated motion patterns are preserved. But such conformational stability must not be interpreted as direct functional inhibition of IL-6 activity. Together, the analyses highlight the role of ligand-induced stabilization in maintaining structural and dynamic integrity of the protein. From a translational perspective, although metformin remains a cornerstone in glycemic control, its limited specificity toward inflammatory cytokines and its well-documented gastrointestinal intolerance and lactic acidosis risk with long-term use highlight the need for safer alternatives.

From a translational perspective, the metabolites identified in this study can serve as preliminary computational leads for further investigation of compounds that are capable of interacting with inflammatory signalling targets such as IL-6. These findings provide an initial structural basis that can be kept as a base for future studies aimed at identifying molecules with potential modulatory effects on inflammatory pathways. While various delivery strategies such as oral formulations in the form of enteric-coated capsules to protect metabolites from gastric degradation, liposomal formulations to enhance intestinal absorption, and probiotic-based delivery systems may be explored in future research, comprehensive *in vitro* validation, *in vivo* studies, pharmacokinetic characterization, and toxicological assessments will be necessary to determine whether these compounds possess meaningful biological activity or therapeutic potential.

Despite the promising findings, it is also very important to acknowledge the limitations of the study. The results are solely based on computational results and do not provide insights on post-translational modifications and protein-protein interactions that occur *in vivo*. Therefore further validation with *in vitro* studies are required to confirm the theraupetic potential of the identified compounds. Without employing *in vitro* validation using appropriate models like insulin-resistant adipocytes, hepatocytes, pancreatic β-cells or macrophage-based culture, the functional aspect and impact on IL-6 expression and inflammatory signalling pathways (e.g., JAK/STAT, NF-κB) cannot be established. Lack of animal model validation limits the understanding of how IL-6 modulation can have an effect on insulin sensitivity, glycemic control and long-term diabetic complications like nephropathy and cardiovascular dysfunction. Finally, absence of dose-response studies and biomarker validation limits the conversion into precision medicine frameworks. The study depended on the GEO2R for the publicly available data which limits control over normalisation procedures and batch effect correction. Furthermore, clinical metadata such as age, sex, BMI and medication status were not fully available in the GEO dataset. These factors may act as potential confounding variables which can influence gene expression. The present study could not explicitly control these but future study including datasets with clinical annotations can further strengthen these findings.

## 5 Conclusion

This study employed an integrative structure-based computational and transcriptomics-driven approach to investigate inflammatory mechanisms associated with T2DM, highlighting IL-6 as a key regulatory hub through differential gene expression and functional enrichment analyses. Considering the limitations of long-term use of conventional antidiabetic and anti-inflammatory drugs, this study explored *B. subtilis*-derived metabolites as potential natural bioactive compounds, supporting the growing interest in microbiome-driven postbiotics. Molecular docking analysis demonstrated that the selected metabolites exhibit moderate binding interactions with key residues of IL-6, suggesting their potential as structural binders for further investigation. Overall, this work provides an *in silico* framework for identifying inflammation-associated therapeutic targets and evaluating natural compounds for metabolic disorders. However, these findings are preliminary and require experimental validation. Collectively, the study offers meaningful insights into the molecular basis of T2DM and supports future research toward microbiome-based interventions targeting inflammation (Ranjani et al., 2026).

## Data Availability

The original contributions presented in the study are included in the article/supplementary material, further inquiries can be directed to the corresponding authors.
